# Genomic mapping of copy number variations influencing immune response in breast cancer

**DOI:** 10.3389/fonc.2022.975437

**Published:** 2022-09-01

**Authors:** Igor López-Cade, Vanesa García-Barberán, Esther Cabañas Morafraile, Cristina Díaz-Tejeiro, Cristina Saiz-Ladera, Adrián Sanvicente, Pedro Pérez Segura, Atanasio Pandiella, Balázs Győrffy, Alberto Ocaña

**Affiliations:** ^1^ Experimental Therapeutics Unit, Hospital Clínico San Carlos (HCSC), Instituto de Investigación Sanitaria San Carlos (IdISSC), Madrid, Spain; ^2^ Molecular Oncology Laboratory, Hospital Clínico San Carlos (HCSC), Instituto de Investigación Sanitaria San Carlos (IdISSC), Madrid, Spain; ^3^ Center for Biological Research, Margarita Salas Centro de Investigaciones Biologicas (CIB)-Consejo Superior de Investigaciones Cientificas (CSIC), Spanish National Research Council, Madrid, Spain; ^4^ Medical Oncology Department, Hospital Clínico San Carlos (HCSC), Instituto de Investigación Sanitaria San Carlos (IdISSC), Madrid, Spain; ^5^ Instituto de Biología Molecular y Celular del Cáncer [IBMCC-Centro de Investigacion del Cancer (CIC)], Instituto de Investigación Biomédica de Salamanca (IBSAL), Consejo Superior de Investigaciones Científicas (CSIC) Salamanca, Salamanca, Spain; ^6^ Centro de Investigación Biomédica en Red en Oncología (CIBERONC), Madrid, Spain; ^7^ Department of Bioinformatics, Semmelweis University, Budapest, Hungary; ^8^ 2^nd^Department of Pediatrics, Semmelweis University, Budapest, Hungary; ^9^ Termeszettudomanyi Kutatokozpont (TTK) Lendület Cancer Biomarker Research Group, Institute of Enzymology, Budapest, Hungary; ^10^ Translational Oncology Laboratory, Translational Research Unit, Albacete University Hospital, Albacete, Spain; ^11^ Centro Regional de Investigaciones Biomédicas, Castilla-La Mancha University (CRIB-UCLM), Albacete, Spain

**Keywords:** breast cancer, CNVs, Gene Amplification, immune response, new surface targets

## Abstract

Identification of genomic alterations that influence the immune response within the tumor microenvironment is mandatory in order to identify druggable vulnerabilities. In this article, by interrogating public genomic datasets we describe copy number variations (CNV) present in breast cancer (BC) tumors and corresponding subtypes, associated with different immune populations. We identified regulatory T-cells associated with the Basal-like subtype, and type 2 T-helper cells with HER2 positive and the luminal subtype. Using gene set enrichment analysis (GSEA) for the Type 2 T-helper cells, the most relevant processes included the ERBB2 signaling pathway and the Fibroblast Growth Factor Receptor (FGFR) signaling pathway, and for CD8+ T-cells, cellular response to growth hormone stimulus or the JAK-STAT signaling pathway. Amplification of *ERBB2, GRB2, GRB7*, and *FGF* receptor genes strongly correlated with the presence of type 2 T helper cells. Finally, only 8 genes were highly upregulated and present in the cellular membrane: *MILR1, ACE, DCSTAMP, SLAMF8, CD160, IL2RA, ICAM2*, and *SLAMF6.* In summary, we described immune populations associated with genomic alterations with different BC subtypes. We observed a clear presence of inhibitory cells, like Tregs or Th2 when specific chromosomic regions were amplified in basal-like or HER2 and luminal groups. Our data support further evaluation of specific therapeutic strategies in specific BC subtypes, like those targeting Tregs in the basal-like subtype.

## Introduction

Cancer is characterized by the presence of modifications in the genomic material that can subsequently lead to a proliferative advantage (1). Alterations in the genomic content like mutations, or copy number variations (CNV) (either amplifications or deletions), can modify cellular functions inducing cell transformation (2). Similarly, translocations can induce cancer, exemplified by the fusion protein BCR-ABL in Chronic Myeloid Leukemia (3, 4).

The identification of these alterations leads to the development of potential therapeutic strategies. For instance, in the case of mutations at the kinase domain, the modified protein can have a hyper-functional activity that could be inhibited by chemical entities acting on the enzymatic area (5, 6). This has been observed with mutations, where a compound was able to compete with the ATP or induce an allosteric change of the kinase pocket (7, 8). Many examples have been described in different solid tumors, and many of these therapies have reached the clinical setting (9–16). Similar findings have been observed with the amplification of genes that code for proteins with a relevant role in cell signaling, like HER2 in BC (17, 18). In this case, targeting the kinase domain of the HER2 protein can augment survival in patients with HER2 positive tumors (19–22). Since very recently, only proteins with an enzymatic activity were able to be inhibited, however, the discovery and development of protein targeting chimeras (PROTACs), has opened the door for other proteins to be degraded, if a chemical compound is able to bind the targeted protein (23). This allows for the first time, the targeting of oncogenic proteins without enzymatic activity, and could be applied for those that are overexpressed secondary to gene amplification (24–26).

BC is a heterogeneous disease, and a particular subtype is that one in which the amplification of HER2 produces a protein overexpression (27–31). Other types of BC include those where the tumorigenesis is led by the presence of the estrogen and progesterone receptors named luminal molecular subtype, or those in which no amplification of HER2 and presence of the estrogen or progesterone receptor exists, this last one is termed triple-negative subtype (32). For all of them, the identification of genomic druggable vulnerabilities is the main objective, as therapeutic options in the metastatic setting are limited (33, 34).

Transformed cells interact with their microenvironment modifying the immunologic response against the tumor (35–37). The presence of tumor genomic alterations can influence the presence of immunologic cells and the effector activity of these cells against the tumor (38). Broadly, it is known that CNV can modulate the efficacy of different anti-cancer agents including chemotherapy as is the case in HER2 overexpressing tumors (39). More and more genomic alterations are known to influence the immune response, which could unmask new treatable and druggable genomic vulnerabilities. For instance, the presence of high mutational burden (HMB), high microsatellite instability (MSI), or deficient mismatch repair (DMMR), are all linked to an increase in genomic instability and a high presence of neo-peptides, which favors an anti-PD-L1 response (40, 41). Although the mentioned alterations are not CNVs, we anticipate that some CNV, which can also be used for therapeutic purposes, could modulate the immune response, offering new insights into the treatment of BC. This will allow us to understand in great detail the underlying mechanisms that modulate the immune response in cancer (42–46).

In our work, we interrogated the BC genome to identify CNV, particularly gene amplification, that could influence the immune response. In addition, we analyzed proteins that were expressed on the cellular surface and that could be therapeutically inhibited with antibodies.

## Material and methods

**Figure 1:**
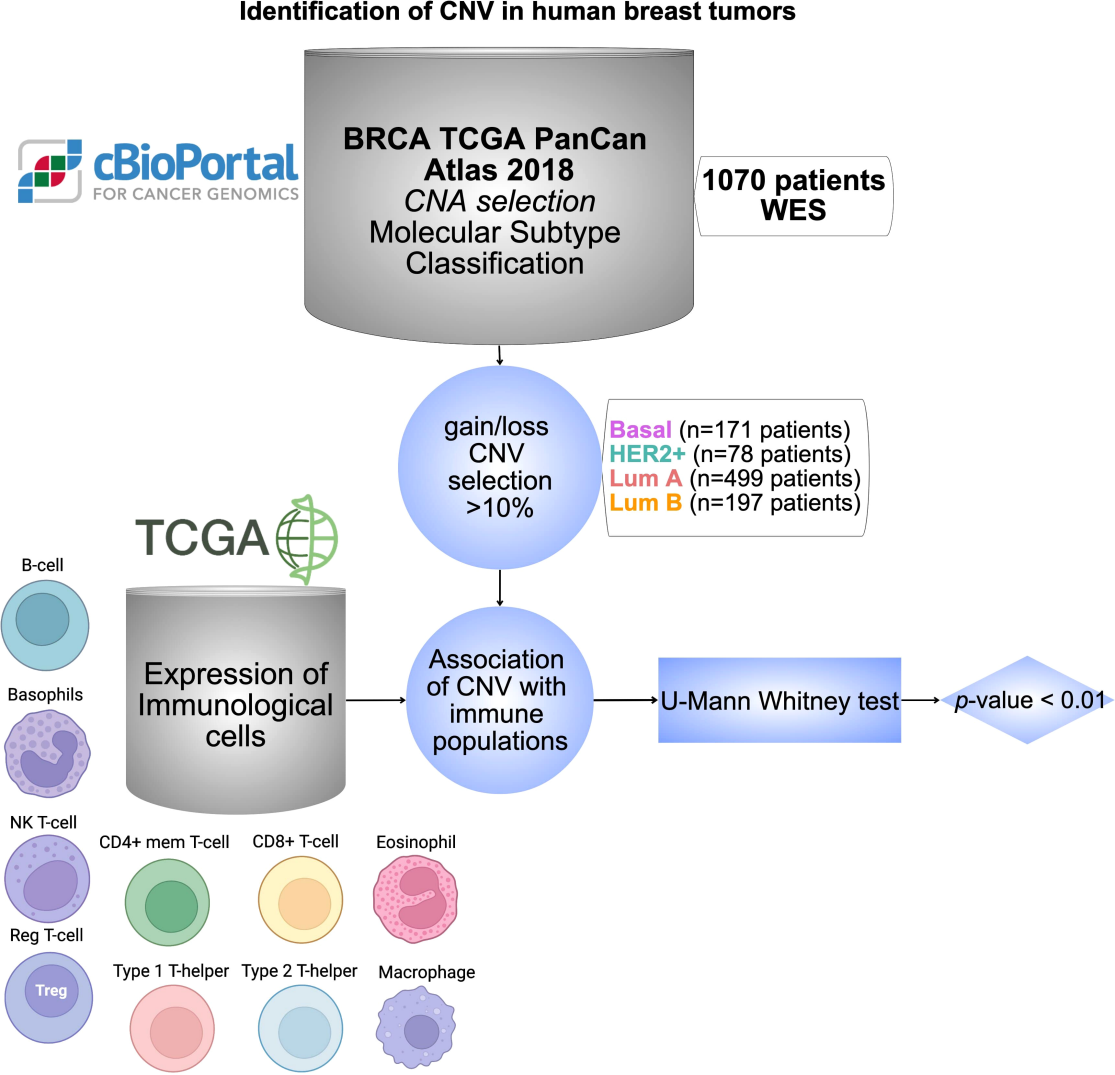
Identification of CNV in human breast tumors. Workflow chart displaying dataset sources and selection criteria of CNV in BC including 1070 patients, and the statistical association with different immune cell populations using TCGA database. Cells were created with BioRender.com.

The global design of the study is displayed in **Figure 1**. Briefly, we have used data from public datasets including TCGA and cBioPortal for cancer genomic analysis. Genes with CNV in more than 10% of the population were selected based on breast cancer subtypes. The analysis of those genes with immune populations was performed later. Finally, a protein-protein interaction analysis and its evaluation with outcome were done for the identified genes by using the String network database (47) and the KM plotter online tool (http://kmplot.com/analysis/), respectively.

### Data origin

Processed TCGA (The Cancer Genome Atlas) PanCancer dataset was obtained from cBioportal (48, 49) (www.cbioportal.org; accessed on December 2019). This dataset data was used to explore genes with CNVs for each molecular subtype of BC. Only genes that were amplificated or deleted in the samples with a frequency higher than 10% were selected.

For the analysis of gene expression and CNVs in clinical samples, we used the entire TCGA BC cohort (50). The gene expression data for each sample was DeSeq normalized in the R statistical environment and a second scaling normalization was executed to set the mean expression across all genes in each sample to 1000 read counts. The gene annotation provided by TCGA was used and the final number of genes in the complete database was 26,272.

### Molecular subtypes

Molecular subtypes were determined based on the St Gallen criteria (51), using the transcriptomic data for each sample. In brief, triple-negative samples lacking HER2, ESR1, and PGR were designated as Basal, HER2 positive and ESR1 negative samples were designated as HER2 enriched, ESR1 positive HER2 negative samples with low KI67 expression were designated as Luminal A, and the remaining samples were assigned to the Luminal B cohort.

### Statistical test

The immunological scores were computed using xCell algorithm (52), which uses the gene expression data as input to compute cell type enrichment scores. The scores ranging between 0 and 1 are representative for the cellular content. The values for each cell type were exported and a filtering was executed so that cell types with a median score below 0.01 were excluded. In addition, only immune cell types were utilized and other cells (e.g. osteoblasts, keratinoblasts, chondrocytes, etc.) were excluded.

Within each molecular subtype, the list of genes was compared to the immune scores (representing the proportion of basophils, B-cells, CD4+, CD8+, eosinophils, macrophages, MS cells, NK cells, regulatory T, type 1 T-cells, type 2 T-cells) between those with depletion or amplification compared to the rest of samples using a Mann-Whitney test. This analysis was performed for each individual gene and we obtained its *p-*value and its fold change (FC). *P-*values below 0.05 were accepted as statistically significant however, we set up a threshold of *p-*value < 0.01 and FC > 1.74 to be more restricted (**Supplemental Data Table 1**). Statistical tests and plots were made using in-home scripts of RStudio. R Core Team (2020). R: A language and environment for statistical computing. R Foundation for Statistical Computing, (Vienna, Austria, https://www.R-project.org/).

### Gene enrichment analysis

The biological process related to each gene set was obtained using the Gene Ontology Biological Process 2021 through the publicly available EnrichR online platform (53–55) (https://maayanlab.cloud/Enrichr/, accessed on: February 2022). The selected genes for this analysis were selected according to each molecular subtype, and the chromosomic regions with more than 10 amplified genes.

Only the top 5 most significant molecular functions were selected for the graphical representation using their negative log in the *p-*values. The full list of molecular functions is saved in **Supplemental Data Table 2**.

### Expression analysis

The analysis comparing the expression level of individual genes between normal breast tissue (*n* = 291) and different BC subtypes (Luminal A, *n* = 415; Luminal B, *n* = 194; HER2, *n* = 66; Basal, *n* = 15) was performed with GTEx and TCGA data using GEPIA2 web server (Gene Expression Profiling Interactive Analysis; http://gepia2.cancer-pku.cn/) (last accessed on June, 2022) (56).

### Identification of surfaceome genes

Protein expression in cell membrane was identified using the Human Surfaceome Atlas (https://wlab.ethz.ch/surfaceome/) (accessed in January, 2022) (57).

The genes that were missing in surfaceome Atlas were consulted on the Genecards website (58), (https://www.genecards.org/, accessed on February 2022)

### Protein-protein interaction

A protein-protein interaction analysis was performed using the String networks database (47) (https://string-db.org/, accessed on July 2022) for the significant genes on the cell surface, where all the information related can be found in the **Supplemental Figure 1**.

### Outcome analysis

The KM Plotter Online tool (59) (https://kmplot.com/analysis/, last accessed on July, 2022) was used to evaluate the relationship between the expression of surfeaceome significantly overexpressed genes and survival in different types of BC (n=2976). This open access database allowed us to investigate Relapse Free Survival (RFS) and Overall Survival (OS). False Discovery Rate (FDR) indicates replicable associations across multiple studies.

## Results

### Identification of CNV in human breast tumors

A total of 25128 genes from 1074 patient samples with BC from the TCGA dataset were evaluated. 171 patients harbored basal-like tumors, 78 HER2+, 499 luminal A, and 197 luminal B BCs. 129 patients could not be classified in any of the mentioned groups as no information from this dataset was available. We selected the CNV for each group that was present in more than 10% of the population. Using this threshold, we identified a total of 1253 amplified genes for the basal-like subgroup, 1648 for the HER2+, 192 for the Luminal A, and finally 685 for the Luminal B subtype. Deletions were observed in 6 genes for the basal-like subgroup, however, for the HER2+, Luminal A, and Luminal B subtypes no deletions were identified. The flow chart describing the process used for the selection of genes and primary sources is shown in **Figure 1** and all information is provided in **Supplemental Data Table 1**.

### Amplified genes are located in specific chromosomal regions

**Figure 2:**
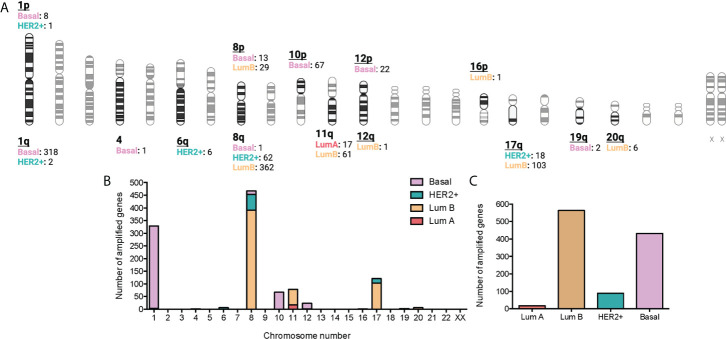
Association between amplified genes and chromosomic location stratified by BC molecular subtype. **(A)**: Chromatogram with amplified gene locations by BC molecular subtype. Created with BioRender.com. **(B)**: Bar graph with the number of gain CNV-related genes by chromosome location and stratified by BC molecular subtype. **C:** Bar graph with the number of gain CNV-related genes by BC molecular subtype.

A *p* < 0.01 was considered significant for the selected associations of CNV genes with immune populations. Some of the amplified genes were located in specific chromosomal regions. For example, at chromosomal 1, region1p, 8 genes were identified for the basal subtype and 1 for the HER2+, and at the region 1q, 318 genes for the basal-like subtype and 2 for the HER2+. Other chromosomes with highly altered genes included the chromosomal 8, with 362 genes in luminal B tumors and 62 genes in HER2+ tumors at the 8q region; 29 in luminal B, and 13 in the basal-like group in the 8p region. Other chromosomes included 11q (61 genes in luminal B and 17 in luminal), and 17q (103 in luminal B and 18 in HER2+). Finally, we identified altered genes only in the basal subgroup (chromosome 4: 1 gene, region 10p: 67 genes, region 12p: 22 genes, and 19q: 2 genes). The chromosome region with more genes altered included the 8q with 425, followed by the 1q with 320 (**Figures 2A, B**). Globally, the tumor type with more amplified genes was the luminal B with 563, followed by the basal-like with 432 and the HER2 with 89, and finally the Luminal A with 17 (**Figure 2C** and **Supplemental Data Table 3**).

### Association of CNV with immune populations

**Figure 3:**
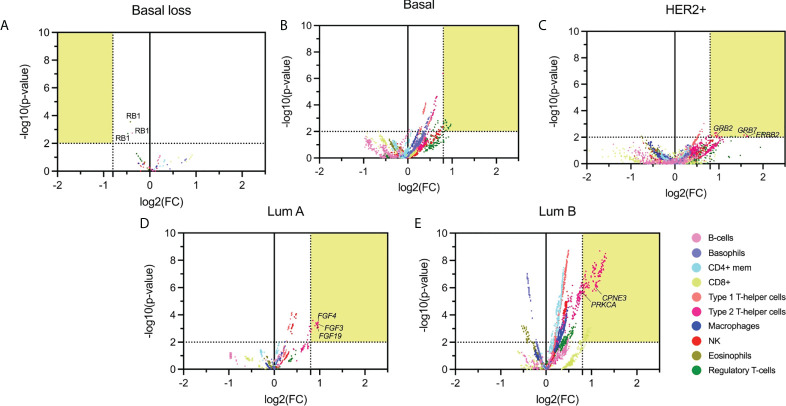
Volcano plot of altered (deleted or amplified) genes with its immune score relationship. The highlighted area corresponds with the thresholds selected parameters for *p-*values lower than 0.01 and FC higher than 1.74. **(A)**: Basal molecular subtype with loss CNV genes; **(B-E)**: Basal, HER2 +, Luminal A, and Luminal B molecular subtype with gain genes. Emphasizing those amplified genes associated with relevant immunological function.

We next correlated the presence of amplified genes with specific immune cell populations using the immune score in each specific BC subtype. The statistical association (*p*-value < 0.01) and FC higher than 1.74 are shown in a volcano plot representation in **Figure 3**. With this approach, we identified 1075 genes in the basal-like subgroup, 1418 in the HER2+, 161 in the Luminal A, and finally 579 in the Luminal B subtype.

Genes amplified in the basal-like subtype were mainly associated with basophils (584 genes), Type 1 T-helper cells (468 genes), and Regulatory T-cells (433 genes), followed by CD8+T cells (286 genes) (**Figure 3B**). In the HER2+ molecular subtype, main amplified genes were associated with Type 1 and Type 2 T-helper cells (199 and 333, respectively), followed by macrophages (103) (**Figure 3C**). In the luminal groups, for luminal A, Type 2 T-helper cells were the principal cell type (141 genes) (**Figure 3D**), and for luminal B (**Figure 3E**), results were in the same direction but with additional cell types: Type 1 T-helper cells (561) and Type 2 T-helper cells (539), followed by CD4+ mem T-cells (531 genes). **Supplemental Data Table 4** describes the whole information concerning amplified genes and immune populations associated with them.

### Gene set enrichment analysis of highly associated genes

**Figure 4:**
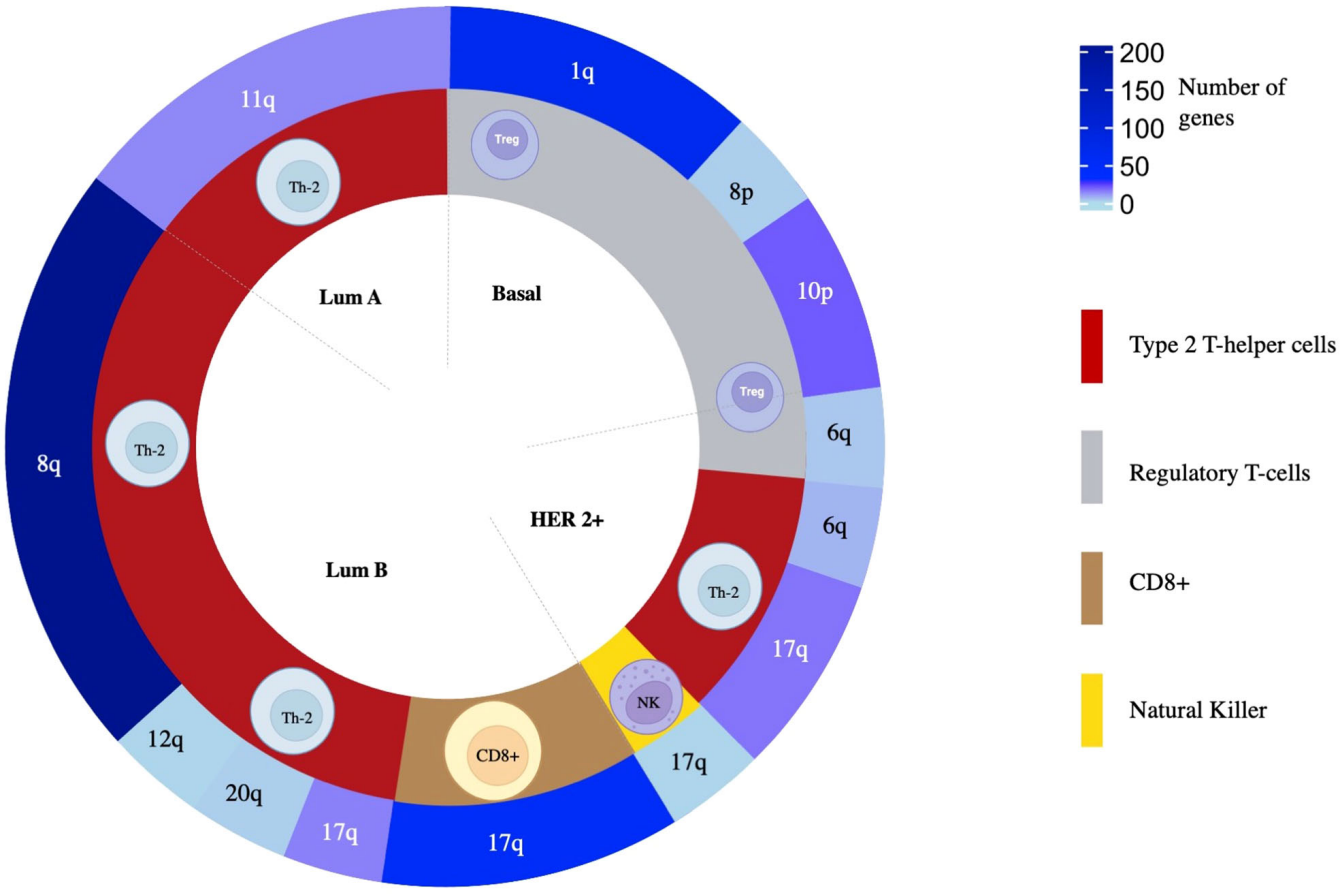
The overall distribution of amplified genes and their association to immune populations. From inside to outside, BC molecular subtype, related immune cell population, and chromosomic location of the genes are colored following a gradient according to the number of amplified genes selected as statistically significant.

Due to the high presence of amplified genes related to different immune populations, we added a more restrictive classification that included only those with a log_2_(FC) greater than 0.8 and a -log_10_(*p*-value) higher than 2 (selected in yellow in **Figure 3**). With these new parameters, in the basal-like subtype, we identified 87 amplified genes included in the Regulatory T-cell population group that was distributed in 65 genes located in chromosome 1q, 2 genes in 8p, and 20 in 10p. In the HER2 + tumors, a total of 26 genes were identified and distributed in 3 immune cell populations with 1 gene located in chromosome 17q for Natural Killer T-cells, 3 genes in 6q for Regulatory T-cells, and 16 and 6 genes in 17q and 6q, respectively for Type 2 T-helper cells. A total of 13 amplified genes were identified for the Luminal A subtype, corresponding to the Type 2 T-helper cell located in 11q. The Luminal B had a total of 292 amplified genes, divided into 48 genes in 17q for CD8+ T-cells. A total of 244 genes for Type 2 T-helper cells were identified and stratified in 227 genes in 8q, 1 gene in 12q, 14 genes in 17q, and 2 genes in 20q, in the mentioned luminal B subtype (**Figure 4**, **Supplemental Data Table 4** **).**

**Figure 5:**
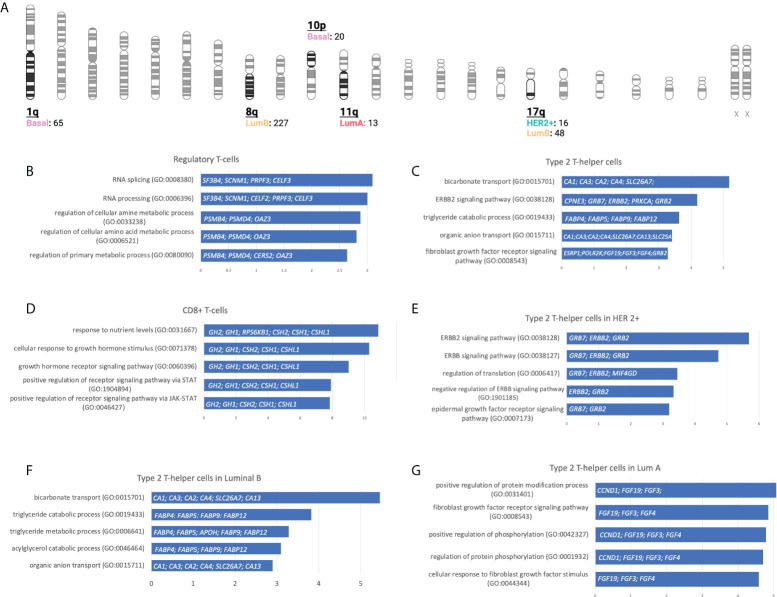
Gene locations and biological processes associated. **(A)**: Number of gain CNV-related genes selected by chromosome location and stratified by BC molecular subtype using gene set enrichment analysis. (Created with BioRender.com). **(B-G)**: Horizontal bar charts with the top 5 Gene Ontology Biological Processes, the genes implicated are located inside the bar plot.

With this information, we performed a gene set enrichment analysis (GSEA) evaluating the top 5 molecular functions ranked by the significance of the *p*-value for transcripts in chromosomic locations that included more than 10 amplified genes (**Figure 5A** and **Supplemental Data Table 4**). The Regulatory T-cells population was associated with the Basal-like subtype, and the most significant molecular process related to genes in chromosome 1q involved RNA splicing and processing (**Figure 5B**). For the Type 2 T-helper cells, the Her2 + tumor and the luminal subtypes were identified, and the most relevant process implicated were the ERBB2 signaling pathway and the Fibroblast Growth Factor Receptor (FGFR) signaling pathway located in chromosomes 8q, 11q, and 17q (**Figure 5C**). For CD8+ T-cells, genes were involved in the cellular response to growth hormone stimulus or the signaling pathway *via* JAK-STAT, and were located in chromosomes 17q (**Figure 5D**).

We next explored GSEA for the type 2 T-helper cells evaluating the difference between the molecular subtypes. For the HER 2 + tumor subtype, all genes implicated were located at chromosome 17q, where we distinguished relevant pathways such as the ERBB2, ERBB, and epidermal growth factor receptor (EGFR) or the negative regulation of ERBB (**Figure 5E**). On the luminal subtypes, we observed processes implicated in organic molecules’ transport or the regulation of the FGFR signaling pathway in Luminal B and A, respectively (**Figures 5F-G**). Indeed, amplifications of *ERBB2, GRB2, GRB7*, and *FGF* receptor genes are strongly correlated with the presence of type 2 T helper cells.

### Some amplified genes code for cell membrane proteins

**Figure 6:**
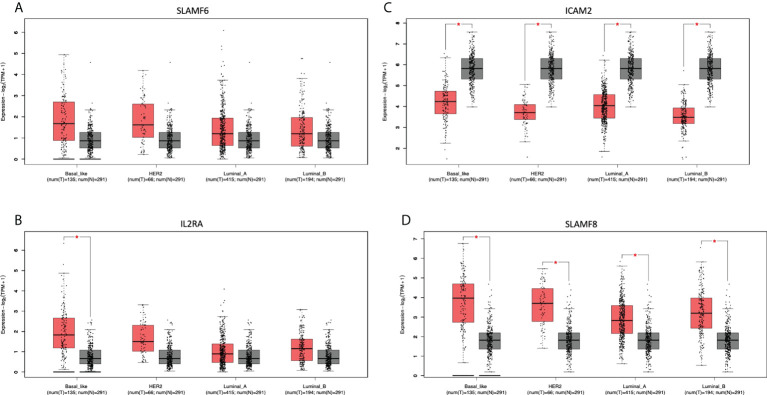
Differential expression in tumoral and normal breast tissue. Gene expression of Tumor (red) vs Normal (gray) samples of **(A)**: *SLAMF6*, **(B)**: *IL2RA*, **(C)**: *ICAM2*, and **(D)**: *SLAMF8*.

We next evaluated which of the identified genes coded for proteins that were located at the cell membrane. Only 8 genes were highly upregulated and present in the membrane: *MILR1, ACE, DCSTAMP, SLAMF8, CD160, IL2RA, ICAM2*, and *SLAMF6*. The function of these genes is described in **Supplemental Data Table 5**. The amplification of these genes in the whole group of breast tumors and by cancer subtype is described in **Supplemental Data Table 6**. Of note, these genes are highly expressed in the majority of tumors as is the case for *SLAMF6* and *SLAMF8* (**Figure 6**), which is correlated with an increase of the CNV amplification in this region.

### Protein-protein interaction, outcome analysis, and potential compounds for the identified genes

We have performed a protein-protein interaction functional enrichment analysis to discover the function of these pathways (47). We have observed a mild correlation coefficient between the genes *CD160, I2RA, SLAMF6, SLAMF8*, and *ICAM2.* While *DCSTAMP, MILR1*, and *ACE* genes show a weak correlation between them **(** **Supplemental Figure 1**).

We have evaluated possible compounds acting on these genes. For *MILR1*, *DCSTAMP*, *SLAMF8*, and *ICAM2*, no targeted drugs were found. However, several drug-targeted genes were identified for the rest of the genes, as displayed in **Supplemental Data Table 7**.

We have performed an analysis evaluating the expression of the identified proteins and patient prognosis including RFS and OS. The outcome analysis reveals that seven genes were associated with outcome, with only five related with detrimental prognosis in OS using RNA-seq database: *ACE, SLAF8, CD160, DCSTAMP*, and *MILR1*, while IL2RA is associated with poor outcome in RFS using gene chip (**Supplemental Data Table 8**).

## Discussion

In the present article, we describe genomic alterations, mainly gene amplifications, associated with an enrichment of immune populations. Beyond the mere presence of a higher mutational load or genomic instability that leads to a higher presence of neo-epitopes and therefore a more immune infiltrate microenvironment (60), a global analysis of copy number variations in relation to immune populations has not been explored. We hypothesized that specific amplifications could be linked to a specific pattern of immune populations within the tumor.

Our first observation was the high association of amplified genes with different cell types, and how these genomic alterations were widely distributed among different regions of the chromosomes. This finding suggests that a wide range of genomic alterations are linked with a host immune response and can potentially contribute to the efficacy of immune oncology agents. In this context, some of them like chromatin remodeling genes, including *SMARC4*, or *ARID1A*, among others, have been associated with efficacy in anti-PD(L)1 (61). In our analysis, two specific subtypes of BC harbored the majority of the amplification associated with immune populations: the luminal B subtype with 563 and the basal-like with 432 CNVs. Of note, in different tumor types, CNV has shown to be linked to outcome as is the case for the amplification of HER2 in a detrimental prognosis in BC (39).

Regarding the specific populations identified in our analysis, the Type 2 T-helper cells, Treg cells, and NK cells were highly associated with the 17q- and 6q-amplified HER2+ molecular subtype, and Type 1 and Type 2 T-helper cells within the luminal A and B subgroup. This was a relevant finding as highlighted the presence of T cell helpers in some specific BC subtypes. It has been extensively reported that tumors where hormones are the main driver of the oncogenic process like luminal A and B BC or prostate cancer display a more immune suppressive microenvironment (62–64). In our case, luminal tumors are associated with the presence of T cell helpers. Th1 and Th2 cell helpers are a subpopulation of immune cells within the CD4+T cell population, that also include Th17, regulatory Tregs (Tregs), and follicular helper (Tfh) (65) cells. Th1 and Th2 have different roles in cancer as Th1 has been proposed to play a role in helping CD8 T cells to kill cancer cells meanwhile Th2 has been associated with tumor promoting involvement (66). Th2 has been associated with the promotion of primary tumors and metastasis by secreting IL-4 and IL-13, which promote M2 tumor associated macrophages (67–69). Interestingly the HER2+ molecular subtype was also associated with the presence of macrophages. In the case of basal-like tumors, the principal cellular subtype was regulatory T cells and included as the principal function RNA splicing and processing. This finding is relevant as Tregs have a clear inhibitory effect on the effector immune response by secreting several cytokines like IL-10, IL-35, or IL-33 (70). In this context RNA splicing and processing suggest the important role that some transcription factors play in the activation and presence of this cellular subtype (71).

When performing gene set enrichment analysis, we observed that two functions, the ERBB2 signaling pathway and the Fibroblast Growth Factor Receptor signaling pathway (FGFR), were highly associated with Type 2 T-helper cells. Specific genes included *ERBB2*, *GRB2*, *GRB7*, and *FGF* receptors. *ERBB2*, *GRB2*, and *GRB7* were highly present in the HER2 positive subgroup and FGF in the luminal A and B. In this regard, activation by membrane receptors tyrosine kinases like ErbB receptors or *FGFR* has been associated with the presence of an immunosuppressive microenvironment and lack of response to anti-PD (L)1 inhibitors (72, 73). In addition, tumor infiltrating lymphocytes (TILs) have been observed in HER2 positive breast tumors (74). HER2 amplification disrupts STING signaling impairing a proper anti-tumor immune response (75). Less evidence associates the expression of FGFR and immune modulation. In one study, FGFR inhibition augmented T cell immune response (76). However, in both cases, a direct correlation of these receptors with Type 2 T-helper populations has not been described.

Finally, we identified some genes that are at the surface of the cell membrane including *MILR1*, *ACE*, *DCSTAMP*, *SLAMF8*, *CD160*, *IL2RA*, *ICAM2*, and *SLAMF6*, and that could be inhibited with therapeutic antibodies. Two of them SLAMF6 and 8 were highly amplified and overexpressed in the majority of tumors. *SLAMF6* is expressed in NK, T cells, B cells, and dendritic cells and some articles have suggested that SLAMF6 plays an inhibitory effect on CD8+ T cells (77). *SLAMF8* has been associated with response to anti PD(L)1 therapies (78). However, the presence of these amplified receptors is mainly located in the membrane of tumoral cells, and their function in tumoral cells is unknown. Other immunologic genes include *CD160* associated with immune escape (79) and *IL2RA* involved in the activation of Tregs (80). Of note, this last finding correlates with the high association of Tregs in the basal-like group.

In summary, we described immune populations associated with genomic alterations with different BC subtypes. We observed a clear presence of inhibitory cells like Tregs or Th2 when specific chromosomic regions are amplified in basal-like or HER2 and luminal groups, respectively. The presence of HER2 and FGRF correlated with Th2. High expression of surface immune genes was identified including *SLAMF6, SLAMF8*, or *IL2RA*. Future studies should be performed to evaluate *in vitro* the function of some of these pathways in the described specific populations and their potential use as targets for therapeutic intervention.

## Data availability statement

The original contributions presented in the study are included in the article/**Supplementary Material**. Further inquiries can be directed to the corresponding author.

## Author contributions

Conceptualization, AO. data curation, BG and IL-C; formal analysis, IL-C, VG-B, and AO; funding acquisition, VG-B and AO; methodology, IL-C and BG; project administration, AO; resources, BG, PPS and VG-B; software, IL-C and BG; supervision, AP, VG-B, ECM and AO; visualization, IL-C, ECM, CD-T, CS-L and AS; writing—original draft, IL-C and AO; writing—review and editing, VG-B and ECM. All authors contributed to the article and approved the submitted version.

## Funding

AO’s lab is supported by the Instituto de Salud Carlos III (ISCIII, PI19/00808). ACEPAIN. CRIS Cancer Foundation and Diputación de Albacete. This research is also supported by PI18/01020from the Instituto de Salud Carlos III and co-financed by the European Development Regional Fund (FEDER) “A way to achieve Europe” (ERDF). EM is supported by a “Juan de la Cierva Incorporación” contract of the Spanish Ministry of Science and Innovation with Ref. IJC2019-041728-I.

## Conflict of interest

AO is a consultant of Servier and a former employee of Symphogen.

The remaining authors declare that the research was conducted in the absence of any commercial or financial relationships that could be construed as a potential conflict of interest.

## Publisher’s note

All claims expressed in this article are solely those of the authors and do not necessarily represent those of their affiliated organizations, or those of the publisher, the editors and the reviewers. Any product that may be evaluated in this article, or claim that may be made by its manufacturer, is not guaranteed or endorsed by the publisher.
