# Supplementary figures and images for: Genomic mapping of copy number variations influencing immune response in breast cancer
Source: Front Oncol. 2022 Sep 1;12:975437. doi: 10.3389/fonc.2022.975437 (PMC9476651; doi:10.3389/fonc.2022.975437)

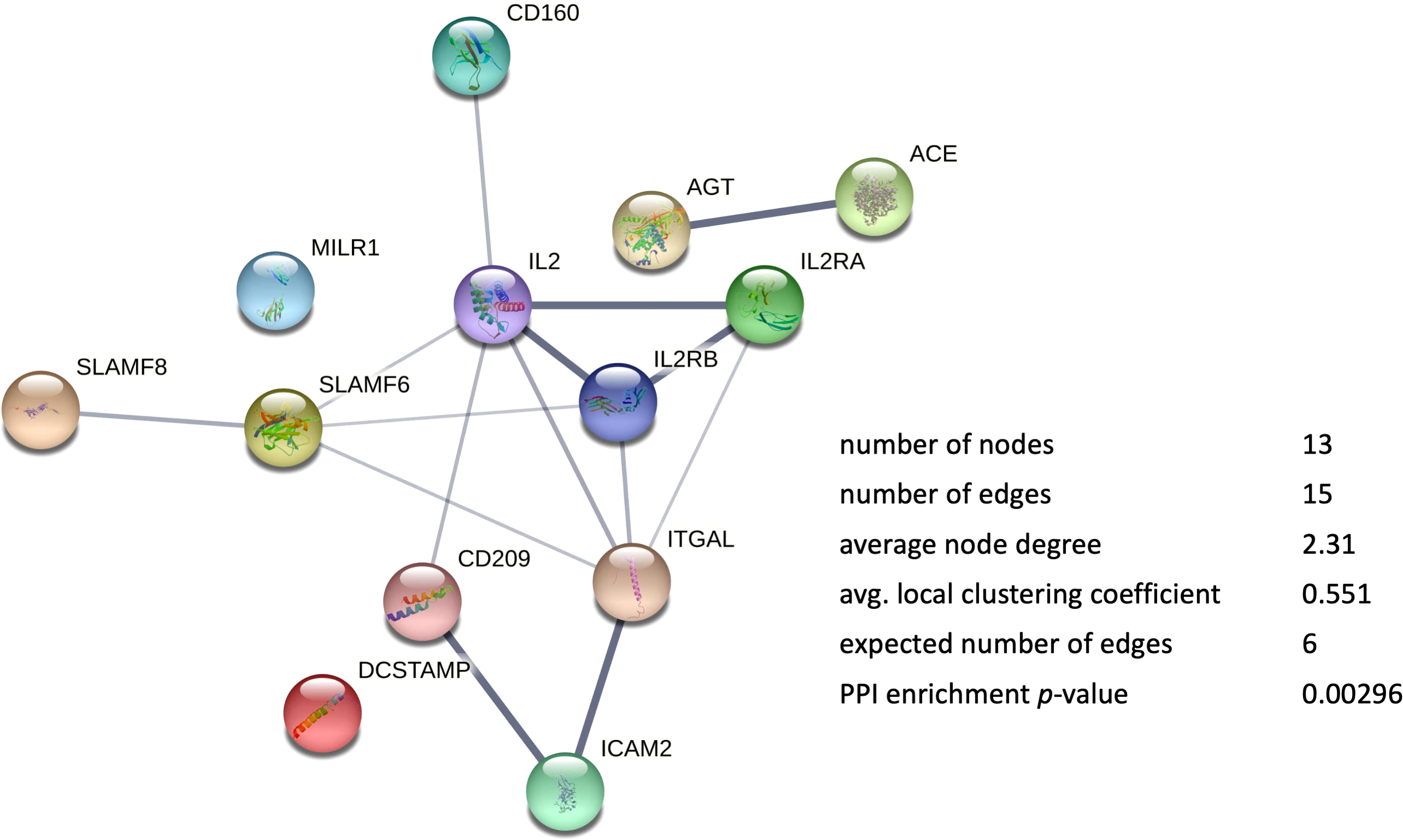

Supplement: Supplementary file 9 [file Image_1.tif]
